# Geometric and dosimetric evaluation of a commercial AI auto‐contouring tool on multiple anatomical sites in CT scans

**DOI:** 10.1002/acm2.70067

**Published:** 2025-03-17

**Authors:** Robert N. Finnegan, Alexandra Quinn, Patrick Horsley, Joseph Chan, Maegan Stewart, Regina Bromley, Jeremy Booth

**Affiliations:** ^1^ Northern Sydney Cancer Centre Royal North Shore Hospital St Leonards New South Wales Australia; ^2^ Institute of Medical Physics University of Sydney Sydney New South Wales Australia; ^3^ Faculty of Medicine and Health University of Sydney Sydney New South Wales Australia

**Keywords:** automatic contouring, artificial intelligence, deep learning, radiotherapy

## Abstract

Current radiotherapy practices rely on manual contouring of CT scans, which is time‐consuming, prone to variability, and requires highly trained experts. There is a need for more efficient and consistent contouring methods. This study evaluated the performance of the Varian Ethos AI auto‐contouring tool to assess its potential integration into clinical workflows. This retrospective study included 223 patients with treatment sites in the pelvis, abdomen, thorax, and head and neck regions. The Ethos AI tool generated auto‐contours on each patients’ pre‐treatment planning CT, and 45 unique structures were included across the study cohort. Multiple measures of geometric similarity were computed, including surface Dice Similarity Coefficient (sDSC) and mean distance to agreement (MDA). Dosimetric concordance was evaluated by comparing mean dose and maximum 2 cm^3^ dose (D_2 cc_) between manual and AI contours. Ethos AI demonstrated high geometric accuracy for well‐defined structures like the bladder, lungs, and femoral heads. Smaller structures and those with less defined boundaries, such as optic nerves and duodenum, showed lower agreement. Over 70% of auto‐contours demonstrated a sDSC > 0.8, and 74% had MDA < 2.5 mm. Geometric accuracy generally correlated with dosimetric concordance, however differences in contour definitions did result in some structures exhibiting dose deviations. The Ethos AI auto‐contouring tool offers promising accuracy and reliability for many anatomical structures, supporting its use in planning workflows. Auto‐contouring errors, although rare, highlight the importance of ongoing QA and expert manual oversight.

## 1 INTRODUCTION

Cancer incidence is increasing globally,^1^ and radiation therapy (RT) provides clinical benefits for about half of all cancer patients.^2^ Current standards of care rely on manual contouring of planning computed tomography (CT) scans to define target volumes and organs at risk (OARs). This process is time‐consuming,^3^ subject to intra‐ and inter‐observer variability,^4^, ^5^ and requires highly trained experts.^6^ As RT technologies advance, there is a growing need for more efficient contouring,^7^ particularly with the uptake of adaptive RT. Automated contouring (auto‐contouring) is needed to provide an effective and scalable method to mitigate these issues,^8^, ^9^ particularly in countries and regions with limited resources.^10^

In recent years, advanced in computing hardware and software have facilitated the development and commercialization of artificial intelligence (AI) auto‐contouring tools.^11^ Studies have highlighted the potential detriment contouring variations have on patient outcomes.^12^, ^13^, ^14^, ^15^ Therefore, a thorough local assessment of any new method of delineation is imperative. Additional considerations when implementing AI auto‐contouring include data privacy, ethical concerns, changes in workflows and workforces, and risk mitigation.^16^, ^17^

Varian Ethos version 2.0 introduced AI auto‐contouring on CT scans. This could facilitate more accurate and consistent online adaptive RT^18^, ^19^ when the same AI models are used for both planning CT and on‐board cone‐beam CT (CBCT).^8^ This study aimed to evaluate the performance of the Ethos AI tool for auto‐contouring of planning CT imaging across various anatomical sites and treatment cohorts to assess the feasibility of implementing this technology within the planning workflow. This study represents the first published assessment of Ethos version 2.0 AI auto‐contouring.

The study objectives were to determine variations in structure definitions between the Ethos AI models and local practice, evaluate auto‐contouring failure modes, measure geometric accuracy and precision of the auto‐contours, and investigate the dosimetric impact of contouring variations.

## 2 METHODS

### 2.1 Patient data

This study used retrospective data from 223 patients treated at Northern Sydney Cancer Centre (Royal North Shore Hospital, St Leonards, Australia) from 2014 to 2020. Inclusion in this study is covered under a local ethics‐approved clinical trial (LNR/15/HAWKE/355). The anatomical sites represented in this cohort included the pelvis, abdomen, thorax, and head and neck regions (Table 1).

**TABLE 1:** Overview of anatomical and treatment sites of patients included in this study.

Anatomical site	Treatment cohort	Number of patients
Pelvis	Anus	24
	Bladder	24
	Gynae	24
Abdomen	Pancreas	24
	Liver (SBRT)	24
Thorax	Oesophagus	24
	Mediastinum	10
	Lung (SBRT)	24
	Breast (left‐sided)	17
Head and neck	Naso‐, oro‐, hypo‐pharynx	28
Total		223

RT simulation scans were acquired on a Philips Brilliant Big Bore CT scanner (Philips Medical Systems, Eindhoven, The Netherlands). All imaging was acquired at 120 kVp in helical scan mode. In‐plane pixel size ranged from 0.59 – 1.17 mm (median 1.17 mm) with slice thickness between 1.5 and 2.5 mm (median 2.0 mm).

#### 2.1.1 Radiotherapy planning

**FIGURE 1:**
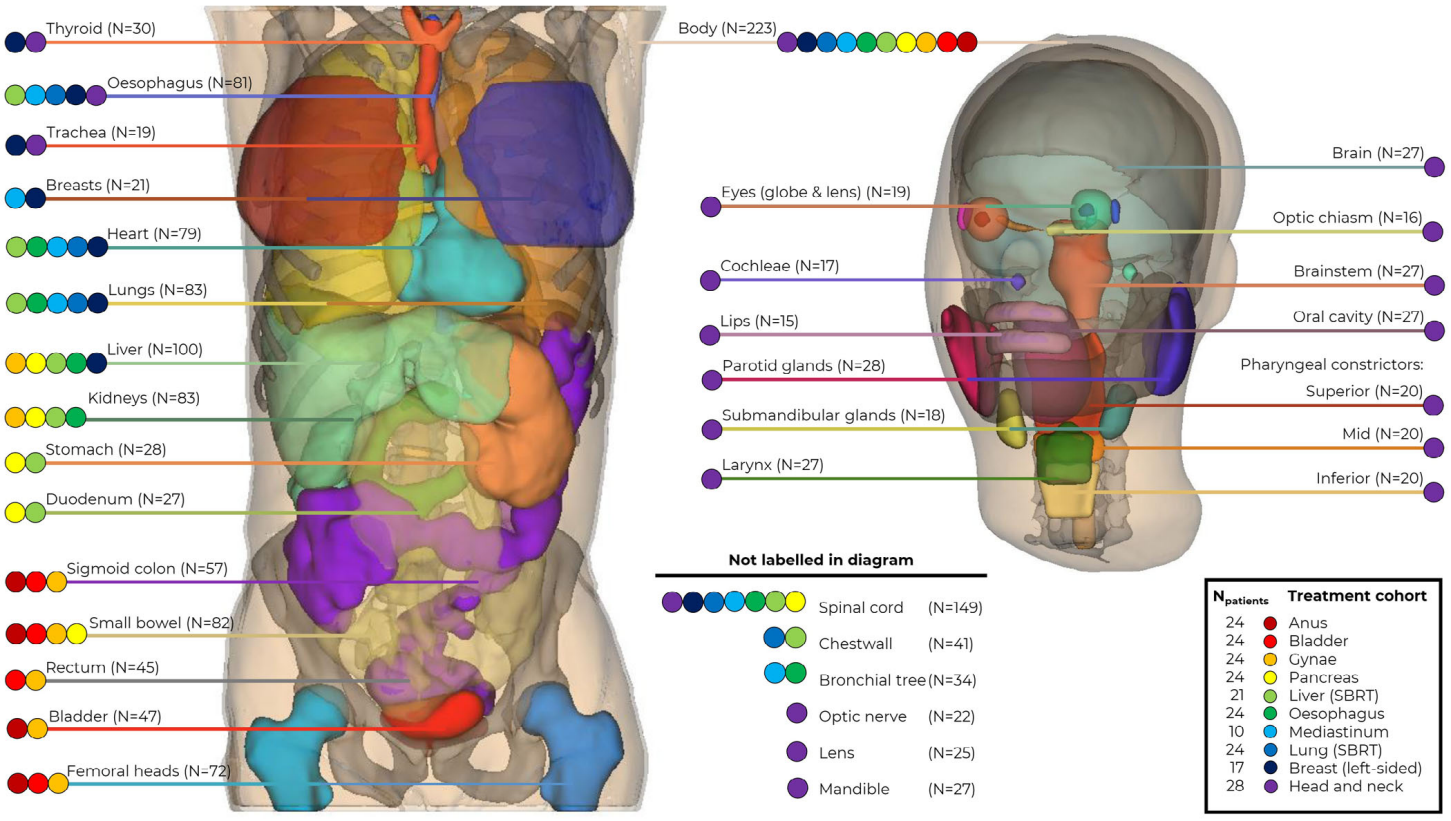
Specific anatomical structures included in this study, with the treatment cohorts for which they can be contoured during radiotherapy planning indicated with coloured circles. The number of cases for which these structures were included is given in parentheses.

For each patient, anatomical and optimization structures were manually contoured on the planning CT images by a radiation therapist or radiation oncologist, according to departmental protocols. The level of experience of those performing contouring varied, however all contours were approved by a senior clinician with 10 or more years of sub‐specialized experience. Among patients within the same treatment cohort, not all patients had the same structures contoured, as the potentially variable location of RT targets determines which OARs are required (Figure 1).

Prescribed doses and fractionation varied across and within treatment site cohorts. Treatments were planned as either intensity modulated radiotherapy (IMRT) or volumetric modulated arc therapy (VMAT) techniques in the Eclipse TPS (Varian Medical Systems, Palo Alto, CA). Patient data, in DICOM format, were anonymized and exported. These included the CT image, structure set, dose grid representing the entire treatment course, and RT plan.

### 2.2 Automatic contouring workflow

Anonymized CT datasets were imported into an Ethos test environment (Varian Medical Systems) for auto‐contouring. The resulting structure sets were exported as DICOM RTSTRUCT files for analysis. Patient DICOM data (CT, dose grid, manual and auto‐contour structure sets) were converted into NIfTI (Neuroimaging Informatics Technology Initiative) format for processing and analysis. The workflow is further described in the following sections.

#### 2.2.1 Deep learning segmentation

The Ethos auto‐contouring tool uses deep learning segmentation models based on convolutional neural networks (CNN). The model weights are static and are not modified while the product is in use nor updated based on input user data.^20^ The CNN architecture, BibNet,^21^ is similar to encoder‐decoder networks like U‐Net^22^ and V‐Net,^23^ but includes interconnections between intermediate network layers to facilitate simultaneous processing of imaging data at multiple resolution levels.

Following inference using the CNN, automated post‐processing within the Ethos treatment planning system (TPS) may involve smoothing, filtering the largest connected component, filling interior holes, and cropping at the superior/inferior limits to avoid tapering. Lastly, overlaps between different structures are corrected.

The training data used to develop the Ethos AI models comprise expertly contoured CT images from multiple clinics in the Americas, Europe, Australia, and Asia.^20^ Structure definitions are largely adopted from published consensus RT contouring guidelines and atlases for OARs.^24^, ^25^, ^26^, ^27^, ^28^, ^29^

The list of contoured structures used in this study are provided in the supplementary material (Table S1). For each patient, only structures with existing manual contours were retained. The external “body” contour was also included despite being semi‐automatically contoured in the Eclipse TPS during RT planning, in order to investigate possible differences to Ethos TPS.

#### 2.2.2 Post‐processing structures with different definitions

Contouring guidelines from various organizations vary in how specific structures are defined.^30^, ^31^ Departmental protocols may simplify or modify these guidelines. It is also common to contour only the part of a structure near the RT target where clinical dose objectives do not require the entire structure to be delineated. As a result, several structures differ in their definition between local practices and Ethos AI (Table S2 in supplementary material). This included use of a bowel bag (local) versus bowel loops (Ethos AI), variations in chest wall thickness (local 2.5 cm, Ethos AI 2.0 cm), and specification of larynx location (local: hyoid to cricoid cartilage, Ethos AI: arytenoid cartilages to thyroid cartilage).

**FIGURE 2:**
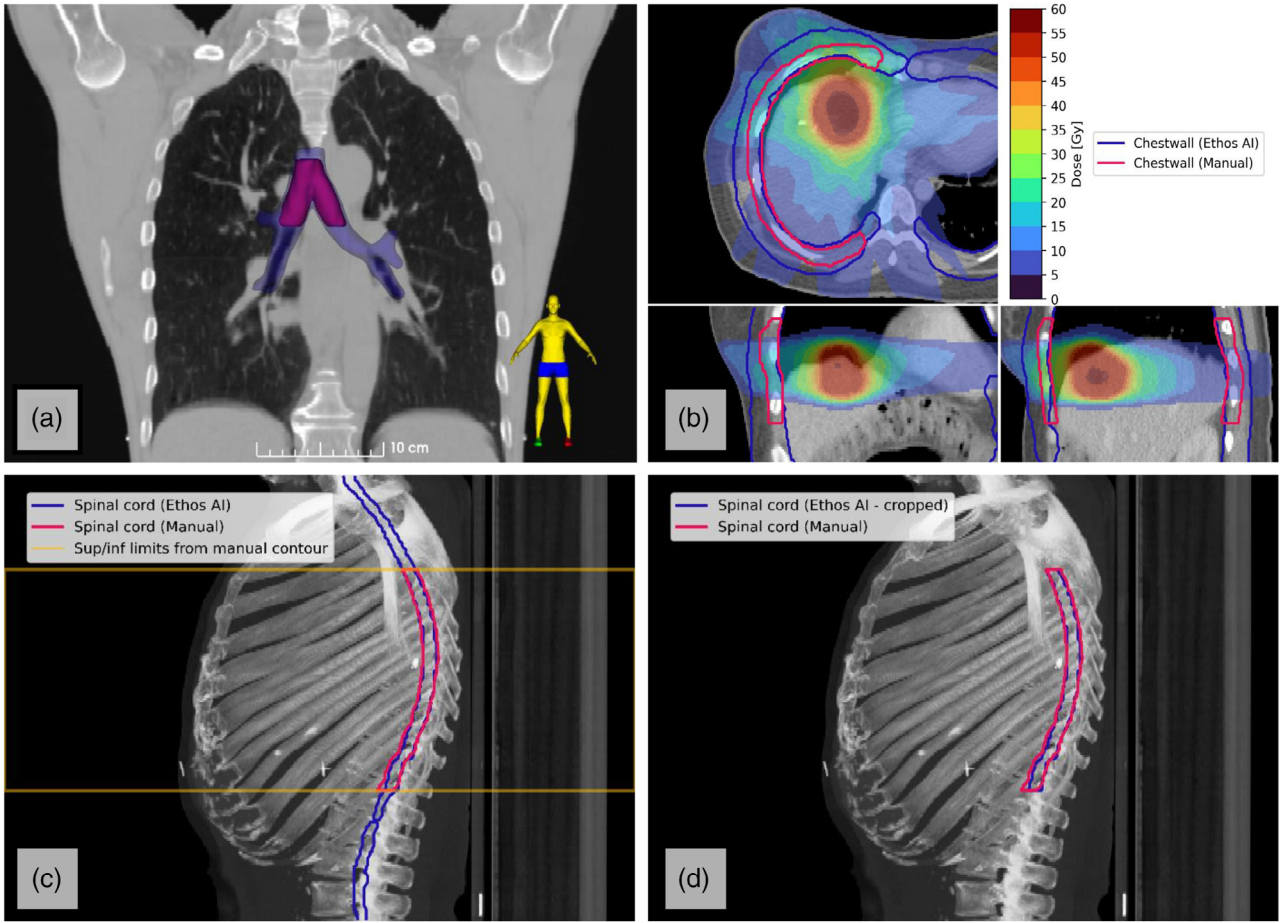
Examples of contouring differences in structure definitions between departmental protocol and the Ethos AI system. (a) The carina (red, manual) is contained within the bronchial tree (blue, Ethos AI), and in this study the relative volume of the carina contained within the bronchial tree was reported. (b) The definition of the chestwall structure differs from departmental protocol and the Ethos AI tool, and the relative volume of the manual contour contained within the auto‐contour was reported in this study. (c and d) Automatic post‐processing of the Ethos AI auto‐contour for the spinal cord incorporated cropping to the superior/inferior limits of the manual contour to enable a fair geometric comparison to manual contours. This process was also applied to the trachea, esophagus, small bowel, and sigmoid colon.

This study aimed to quantitatively assess how adopting different structure definitions might impact departmental processes and establish a baseline for evaluating other available AI auto‐contouring tools. Automatic post‐processing pipelines were designed to address these differences (Table S2, Figure 2). For some structures (i.e., small bowel, sigmoid colon, bronchial tree), differences in definitions preclude assessment using standard geometric measures of similarity, so volume overlap was reported instead.

Post‐processing was used solely for geometric analysis; dosimetric analysis was conducted directly on the auto‐contours from the Ethos test environment.

### 2.3 Visual, geometric, and dosimetric assessment

Each case was visually assessed for large discrepancies between manual and Ethos AI contours, to identify auto‐contouring failures or non‐contiguous volumes, and to evaluate overall qualitative similarity in contours. This process was performed by inspecting axial, sagittal, and coronal slices of the CT scan with both manual and Ethos AI contours overlaid, as well maximum intensity projections along each axis with the projected outline of the contours overlaid.

The performance of the Ethos AI auto‐contouring tool was evaluated using several complementary geometric similarity metrics,^32^, ^33^ implemented in the PlatiPy open‐source software library.^34^ These include the Dice Similarity Coefficient (DSC)^35^ which measures the spatial overlap between two contours, ranging from 0 (no overlap) to 1 (perfect overlap). Although widely reported and easy to interpret, the DSC is highly correlated with the physical size of the delineated structure. The surface DSC (sDSC)^36^ quantifies boundary overlap within a specified level of agreement (denoted *τ*), chosen in this work to be 3 mm, and overcomes the main limitation of the DSC. The mean distance to agreement (MDA)^37^ and Hausdorff distance (HD)^38^ measure average and maximum 3D boundary separations, respectively. Lastly, the volume ratio was reported to capture systematic differences in delineations.

The clinical impact of spatial variations in contouring depends on the dose distribution, the specific structures and associated clinical objectives, and the nature of the spatial variations.^33^ The mean dose (*D*_mean_) and maximum dose delivered to 2 cm^3^ (*D*_2 cc_) were calculated for manual and unedited Ethos AI contours. These metrics were selected to provide complementary information, presenting both a volume‐dependent (*D*_mean_) and volume‐independent (*D*_2 cc_) dose measurement, as well as capturing differences over the entire structure (*D*_mean_) and at high‐dose regions (*D*_2 cc_).

The level of dosimetric concordance between manual and auto‐contours was assessed by computing several measures, all implemented using Scikit‐Learn^39^:

- Pearson correlation coefficient (*r*)

A measure of the linear correlation between the doses calculated from manual and Ethos AI contours, providing a quantitative value that ranges from 0 (no linear correlation) to 1 (perfect positive linear correlation).

- Average error (Avg Err [Gy])

The mean difference in a specific dose metric between the manual and Ethos AI contours, calculated across patients. This metric helps in understanding the typical deviation one might expect when using automated contours.

- Maximum error (Max Err [Gy])

The Max Err identifies the largest single dose metric discrepancy observed between the manual and Ethos AI auto‐contours across patients. This measure highlights the worst‐case scenario in dose variations due to contouring differences.

- Mean absolute percentage error (MAPE)

The MAPE expresses the average absolute relative error between the doses calculated from manual versus Ethos AI auto‐contours, providing an assessment of the variation among patients. This is also useful for comparing the accuracy of Ethos AI contours across different dose ranges, structures, and prescriptions.

## 3 RESULTS

### 3.1 Automatic contouring workflow

**FIGURE 3:**
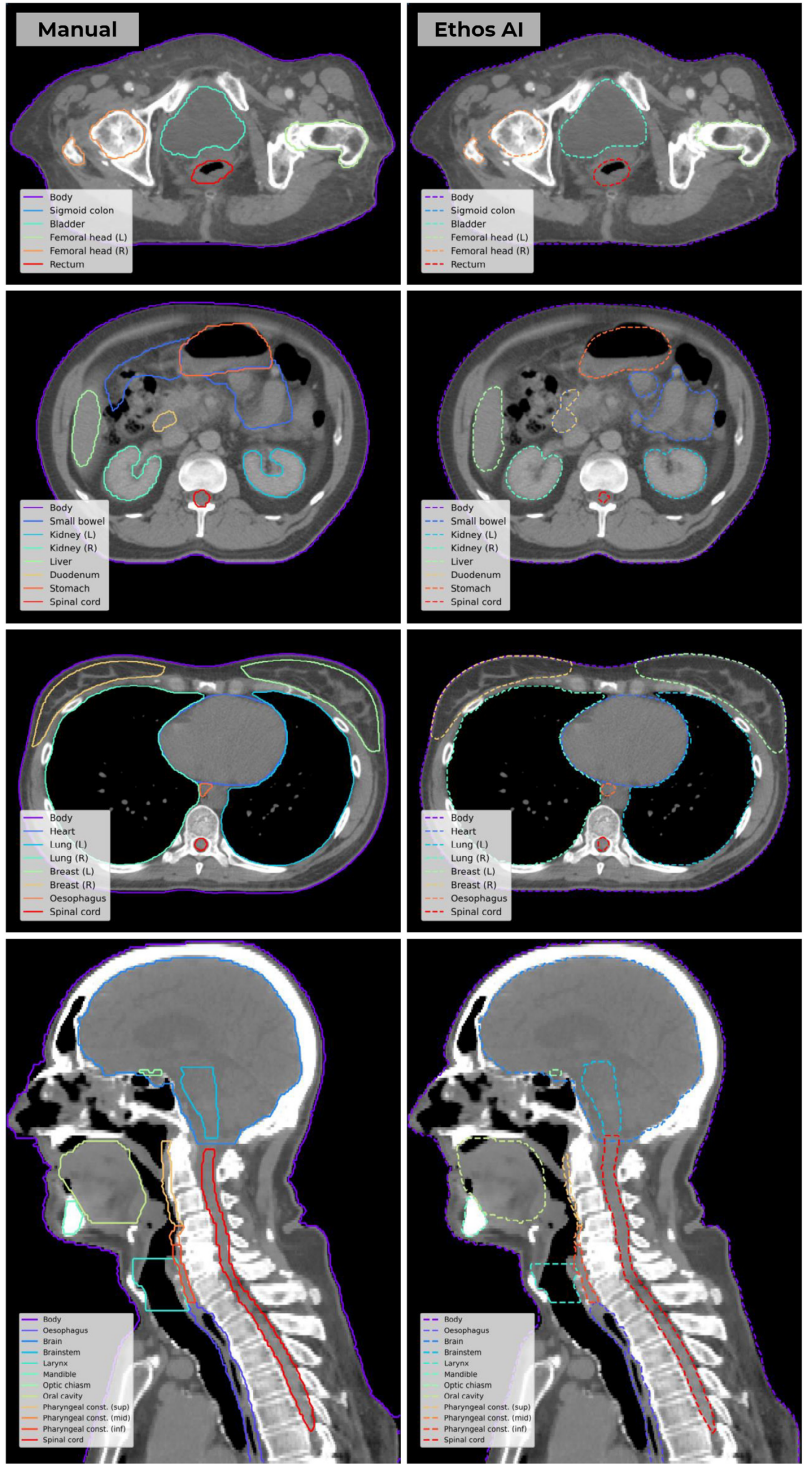
Representative examples from the pelvis (top row), abdomen (second row), thorax (third row), and head and neck (bottom row) cohorts, demonstrating the performance of Ethos AI auto‐contouring.

**FIGURE 4:**
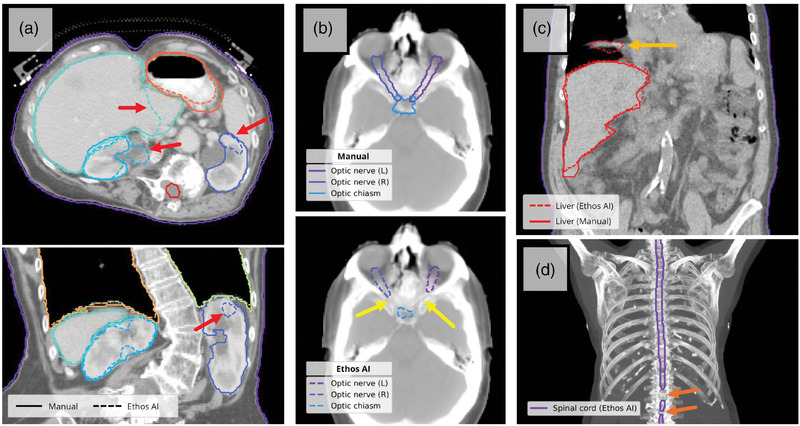
Four representative examples demonstrating contouring discrepancies and errors observed using Ethos AI auto‐contouring, indicated in each case with arrows. (a) Atypical anatomy (severe scoliosis) with auto‐contouring deviations of the kidneys, especially left kidney, and liver. (b) Under‐contouring of the optic nerves and chiasm. (c) Image artifact from breathing motion associated with auto‐contouring deviation. (d) Non‐contiguous spinal cord volume.

Visual assessment showed that Ethos AI models successfully generated auto‐contours for most patients and structures. Representative examples are presented in Figure 3. Greater agreement between automatic and manual contours was seen for clearly demarcated structures such as the bladder, femoral heads, and lungs. Structures that exhibited reduced agreement typically had less well‐defined start/end points, such as the rectum and duodenum, although consistent differences in structure definitions was observed for the breasts which also resulted in poor agreement between manual and automatic contours. Few auto‐contouring errors were observed (see Figure 4), including non‐contiguous volumes and large deviations from patient anatomy.

### 3.2 Visual, geometric and dosimetric assessment

**FIGURE 5:**
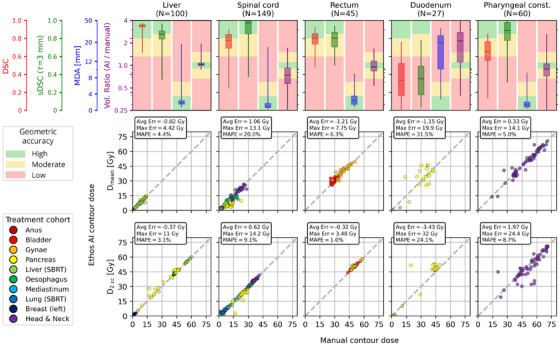
Summary of geometric (top row) and dosimetric (middle, bottom rows) results aggregated over the entire study cohort for five representative structures. The color scales for measures of geometric similarity are a visual guide for geometric auto‐contouring accuracy, with thresholds indicating high (green), moderate (yellow), and red (poor) accuracy for each metric.

Auto‐contouring accuracy varied considerably between structures, which is highlighted for several structures in Figure 5 (complete data provided in supplementary material, Figure S1). Ethos AI demonstrates excellent agreement with manual contouring for the bladder, femoral heads, kidneys, liver, lungs, thyroid, and mandible with mean sDSC ranging from 0.82 to 0.97 and mean volume ratios within 0.93–1.16. Structures such as the rectum, stomach, and submandibular glands were delineated with moderate accuracy (mean sDSC 0.65–0.93, mean volume ratios 0.89–1.45), while the breasts, heart, trachea, and spinal cord showed moderate accuracy with some systematic variations (mean sDSC 0.65–0.94, mean volume ratios 0.76–1.56). The duodenum exhibited notably poor geometric agreement (mean sDSC = 0.32, mean volume ratio = 2.8).

Differences in similarity metrics provided additional insight into performance. A lower sDSC than DSC indicated typical boundary separations greater than 3 mm, as seen in breast contours. Conversely, a higher sDSC than DSC suggested surface variations of less than 3 mm, as with the thyroid, esophagus, trachea, and spinal cord. Overall, 70% of all auto‐contoured volumes had a sDSC > 0.8, 74% achieved an MDA < 2.5 mm, and 56% had an HD < 12.5 mm (values representing a high level of agreement for most structures).

Outliers were identified based on atypical similarity metrics. Auto‐contour failures, defined as a contour including a considerable portion of incorrect anatomical structures or missing large regions of the structure of interest, often occurred in cases where patients had atypical anatomy (Figure 4a), which can be expected due to out‐of‐distribution data.^40^ Failures were included regardless of the suspected cause (e.g., atypical anatomy, image artefacts). The spinal cord was most affected by auto‐contouring failures (*N* = 5), followed by the heart, liver, bladder, and kidney (each *N* = 2). Clinical variations in manual contouring, such as delineation of only a region of the structure, or inclusion of neighboring OARs within a structure, also contributed to geometric dissimilarity (see Figure S2, demonstrating a case where the manual esophagus contour includes the proximal stomach). Differences in external body contours were typically due to the inclusion of items like the couch or immobilization devices (see Figure S3, showing a case in which the “manual” body contour included an abdominal compression plate).

Levels of concordance between *D*_mean_ and *D*_2 cc_ varied by structure and treatment site (Figure 5, complete results in the Supplementary material Figures S4, S5). For some structures, such as the liver, high geometric accuracy translated to excellent concordance in dose metrics with a MAPE of 4.3% and 3.2% for *D*_mean_ and *D*_2 cc_, respectively. Although the spinal cord, rectum, and pharyngeal constrictor muscles were auto‐contoured with similar accuracy (sDSC = 0.89 ± 0.14, 0.78 ± 0.11, 0.86 ± 0.15, respectively), the dosimetric consistency was varied considerably, with MAPE for *D*_2 cc_ of 8.5%, 1.0%, and 8.7%, respectively. Duodenum contours, with noted poor auto‐contouring accuracy, had good dosimetric consistency, although notable outliers contributed to large maximum errors in both *D*_mean_ (Max Err = 19.9 Gy) and *D*_2 cc_ (Max Err = 32.0 Gy).

Variations in structure definitions between Ethos AI and local practice led to discrepancies in dosimetric agreement, especially in mean dose, which depends on structure volume. This was evident for the chestwall; while *D*_2 cc_ showed good agreement (MAPE = 5.6%, Avg Err = −0.57 Gy), the *D*_mean_ did not (MAPE = 72%, Avg Err = 5.22 Gy).

*D*_2 cc_ for small bowel and sigmoid colon auto‐contours was highly concordant with manual contours, with MAPE of 4.6% and 3.4%, respectively, despite considerable differences in definitions. However, certain cases showed greater differences with reported Max Err of 31.88 Gy and 17.11 Gy, respectively, reflecting variations in relative volume overlap (mean ± standard deviation of 88.8% ± 11.9%, see Supplementary material Figure S6). The impact of auto‐contouring variations depended on treatment site and planning strategies, exemplified with more consistent dose metrics (high correlation and low error) for the stomach and duodenum contours in liver SBRT plans compared with pancreas RT plans.

## 4 DISCUSSION

This study presents an extensive assessment of the Ethos AI auto‐contouring tool, highlighting its promise in reliably generating accurate delineations for OARs, particularly for well‐defined and less complex anatomical structures. This underpins the capacity of Ethos AI auto‐contouring to integrate with existing clinical workflows and enhance the efficiency of RT planning. A unique aspect of this study is the evaluation across a wide range of treatment sites and patient cohorts using routine, real‐world data, which captures clinical variations and provides useful benchmarks.

A large proportion of Ethos AI auto‐contours achieved a high level of consistency to manually‐defined reference delineations, though the results of this work emphasize the importance of presenting multiple complementary measures of geometric and dosimetric accuracy. This approach enabled a more detailed characterization of the Ethos AI auto‐contouring tool, especially given the variable size and complexity of structures. While the number of auto‐contouring failures of the Ethos AI system was low and was often associated with atypical patient anatomy or apparent image artifacts, the ongoing need for expert manual oversight is stressed. This is particularly important given auto‐contouring failures often affected critical structures such as the spinal cord and optic nerves. Some differences in structure definitions were identified, which must also be considered if auto‐contours are to be used clinically.

Direct comparisons to other studies are challenging due to variations in patient data, contouring practices, study design, and analysis methods. Most published research on auto‐contouring focuses on one or few anatomical sites, limiting the assessment of an AI tool's overall utility.^41^ However, many studies investigating both in‐house and commercial AI auto‐contouring tools report results consistent with this study's findings.^42^, ^43^, ^44^, ^45^, ^46^, ^47^, ^48^, ^49^, ^50^, ^51^, ^52^, ^53^, ^54^, ^55^, ^56^, ^57^, ^58^, ^59^, ^60^, ^61^, ^62^

Typical accuracy for pelvic structure aligns with reported DSC: 0.80–0.95 for the bladder, 0.70–0.87 for the rectum, and 0.80–0.98 for femoral heads (this work: 0.94 ± 0.04, 0.79 ± 0.09, 0.95 ± 0.02, respectively).^42^, ^43^, ^44^, ^45^, ^46^, ^47^, ^48^, ^49^ In the abdomen, findings of this work are also corroborated by other studies,^42^, ^43^, ^50^, ^51^, ^52^ although reduced auto‐contouring accuracy for the kidneys in this work (DSC = 0.91 ± 0.08, compared with 0.95–0.96^42,43^) may be impacted by several outliers representing low quality and failed auto‐contours. This discrepancy may also be due to the use of in‐house data for both model training and testing in one study,^43^ and small study cohort (*N* = 15) in another^42^, which may not include patients with atypical anatomy or image artefacts, which were present in this work. Accuracy of auto‐contouring for thoracic structures has been the focus of a number of recent studies,^42^, ^43^, ^44^, ^45^, ^46^, ^50^, ^53^, ^54^, ^55^, ^56^ which report typical DSC values ranging from 0.75 (esophagus) to 0.98 (lungs), matching findings of this work: 0.75 ± 0.12 for esophagus and 0.97 ± 0.01 for lungs. Discrepancies are noted for the thyroid, for which Ethos AI achieved a DSC = 0.63 ± 0.18, compared to 0.75–0.88 in other studies.^43^, ^44^, ^53^ For structures in the head and neck region, results from this work generally agree with published data.^57^, ^58^, ^59^, ^60^ Structures that are larger or have clearly defined boundaries (e.g., brain, parotid glands, mandible) are most accurately auto‐contoured, while smaller, less well‐demarcated structures (e.g., optic nerves, optic chiasm, pharyngeal constrictor muscles) tend to be subject to reduced auto‐contouring accuracy. Additional data can be obtained from recent reviews.^61^, ^62^

The dosimetric component of our assessment of Ethos AI auto‐contouring provides insight into how geometric discrepancies translate into variations in *D*_mean_ and *D*_2 cc_. In general, large well‐defined structures (e.g., liver, lungs), tended to exhibit higher geometric accuracy which corresponded to lower mean absolute percentage errors in both *D*_mean_ and *D*_2 cc_. Conversely, for smaller or more irregular OARs (e.g., brainstem, cochleae) minor boundary variations were more likely to result in larger deviations in both dose metrics. Systematic contouring differences were also reflected in dose metrics for some structures (e.g., breast, larynx), noting exceptions for comparisons of *D*_2 cc_ between manual and Ethos AI contours for the small bowel and sigmoid colon, which had reasonable agreement (MAPE = 4.6% and 3.4%, respectively) despite different contour definitions. The clinical significance of variations in dose metrics depends on several factors, including the OAR tolerance, prescription dose, treatment intent, and patient‐specific considerations. A small absolute difference in dose could be critical for radiation‐sensitive OARs close to tolerance, whereas the same difference may be less significant in other contexts. Although this study focused on systematically quantifying these variations rather than establishing thresholds for clinical acceptability, these results highlight the importance of thorough investigation into the impact of AI contour on dose‐volume metrics prior to implementation. Additional prospective work that investigates the relationship between these dosimetric differences and clinical outcomes (e.g., toxicity or tumor control) will be crucial to guide evidence‐based thresholds for auto‐contouring accuracy.

This study has several limitations. First, its retrospective nature means that manual contours were selected from clinical planning data, potentially introducing unwanted variations based on patient‐specific factors, planning practices, and changes over time which may impact the results. However, this approach ensured that manual contouring was performed without potential bias from knowledge of involvement in this study. The use of a single set of reference contours per patient in this work means that it is not possible to measure manual inter‐observer contouring variability, which could provide patient‐specific baselines of achievable precision to assess AI auto‐contours. As clinicians from different specialties generated the clinical contours there is potential for additional variability due to dissimilarities in contouring practices between site specialties. These factors increase the uncertainty in evaluating the performance of the Ethos AI auto‐contouring tool, as some differences between AI‐generated and manual contours reflect these additional sources of contouring variability. There is a growing body of work on inter‐observer contouring variability for radiotherapy volumes.^63^ Reported levels of geometric consistency for expert manual contouring is largely similar to the accuracy of Ethos AI auto‐contouring for structures in the pelvis and lower abdomen,^64^, ^65^, ^66^, ^67^ thorax and upper abdomen,^68^, ^69^, ^70^, ^71^, ^72^ and HN regions.^24^, ^59^, ^73^, ^74^, ^75^ Additionally, this study did not consider auto‐contouring of target volumes, even when these might be considered as anatomical structures (e.g., prostate). Target delineation often incorporates clinical knowledge and is informed by local departmental practice and patient factors not available from the planning CT image alone.

Manual delineations, although created by trained experts, are known to exhibit potentially considerable inter‐observer variability.^4^ In this respect, auto‐contouring has the potential to not only reduce the time needed to contour a patient image,^76^ but also improve contouring and planning consistency.^77^ Further work is needed to verify these hypotheses for the Ethos AI auto‐contouring tool. Additionally, dosimetric data presented in this study was extracted from the clinical plan, optimized with manual contours. End‐to‐end testing of Ethos AI contouring requires comparisons of plans generated using these contours.

As noted above, differences in contour definitions between current clinical practice and Ethos AI models may influence treatment planning.^78^, ^79^ Practically, this may require changes to both dose‐volume constraints and optimization objectives. Dose−volume constraints based on auto‐contours may be assessed by using existing acceptable radiotherapy plans and calculating dose‐volume metrics for auto‐contours, in a process similar to the dosimetric assessment performed in this study. For structures with unacceptable variation in dose‐volume metrics, for example due to differences in structure definitions, revision of constraints may be necessary. Use of AI auto‐contours for inverse optimization implies changes to objectives may also be needed in order to generate treatment plans consistent with current clinical practice, which may require iterative testing prior to clinical implementation of AI auto‐contours and ongoing monitoring following adoption.^16^

The current study serves an important role to identify which structures are suitable for use in planning without modification, and which require potential changes to clinical contouring practices and dose constraints to use auto‐contours based on different definitions. In shifting towards integration of auto‐contouring, additional quality assurance (QA) is necessary to identify segmentation errors, potentially using a second, independent auto‐contouring framework.^80^ The benchmark of delineation accuracy as determined in this current study could be used to inform thresholds on geometric consistency to automate part of this QA.^81^, ^82^ Lastly, a primary driver for automatic contouring is the broader adoption of online adaptive RT. The Ethos platform is designed around on‐board kV‐CBCT imaging acquired immediately prior to adaptive treatment,^18^, ^19^ and further testing of auto‐contouring performance in this context is planned.

## 5 CONCLUSION

Ethos AI auto‐contouring demonstrates accurate and reliable contouring for many structures, showing promise for a large number of OARs across a range of anatomical sites. A comprehensive geometric analysis provides valuable data for future studies. Dosimetric analysis was used to further understand potential clinical impacts of auto‐contouring variations. Observed auto‐contouring discrepancies and failures, although infrequent, highlight the need for ongoing expert oversight. Further work to investigate the clinical adoption of this technology is warranted.

## AUTHOR CONTRIBUTIONS

Robert N. Finnegan, Alexandra Quinn, Regina Bromley, and Jeremy Booth designed the study. Data processing and analysis was performed by Robert N. Finnegan. Alexandra Quinn, Patrick Horsley, Joseph Chan, Maegan Stewart, Regina Bromley, and Jeremy Booth participated in interpretation of data. Maegan Stewart, Regina Bromley, and Jeremy Booth provided project support. All authors reviewed the manuscript and provided feedback on the findings.

## CONFLICT OF INTEREST STATEMENT

R.F., J.B. report a relationship with SeeTreat Pty., Ltd. that includes consulting or advisory services. There are no additional conflicts of interest to disclose.

## ETHICS STATEMENT

Inclusion in this study is covered under the OPT OUT clinical trial, approved by the local ethics review board (LNR/15/HAWKE/355).

## Supporting information



Supporting information

## Data Availability

Data supporting the findings of this study will be made available upon reasonable request to the corresponding author.

## References

[1] Bray F , Laversanne M , Sung H , et al. Global cancer statistics 2022: GLOBOCAN estimates of incidence and mortality worldwide for 36 cancers in 185 countries. CA Cancer J Clin. 2024;74(3):229‐263. 10.3322/caac.21834 38572751

[2] Delaney G , Jacob S , Featherstone C , Barton M . The role of radiotherapy in cancer treatment: estimating optimal utilization from a review of evidence‐based clinical guidelines. Cancer. 2005;104:1129‐1137. 16080176 10.1002/cncr.21324

[3] Montague E , Roques T , Spencer K , Burnett A , Lourenco J , Thorp N . How long does contouring really take? Results of the royal college of radiologists contouring surveys. Clin Oncol. 2024;6:335‐342. 10.1016/j.clon.2024.03.005 38519383

[4] Vinod SK , Jameson MG , Min M , Holloway LC . Uncertainties in volume delineation in radiation oncology: a systematic review and recommendations for future studies. Radiother Oncol. 2016;121:169‐179. 27729166 10.1016/j.radonc.2016.09.009

[5] Vinod SK , Min M , Jameson MG , Holloway LC . A review of interventions to reduce inter‐observer variability in volume delineation in radiation oncology. J Med Imaging Radiat Oncol. 2016;60:393‐406. 27170216 10.1111/1754-9485.12462

[6] Cacicedo J , Navarro‐Martin A , Gonzalez‐Larragan S , De Bari B , Salem A , Dahele M . Systematic review of educational interventions to improve contouring in radiotherapy. Radiother Oncol. 2020;144:86‐92. 31786422 10.1016/j.radonc.2019.11.004

[7] Van Dyk J . The Modern Technology of Radiation Oncology: A Compendium for Medical Physicists and Radiation Oncologists. Medical Physics Publishing; 1999.

[8] Huynh E , Hosny A , Guthier C , et al. Artificial intelligence in radiation oncology. Nat Rev Clin Oncol. 2020;17:771‐781. 32843739 10.1038/s41571-020-0417-8

[9] Parkinson C , Matthams C , Foley K , Spezi E . Artificial intelligence in radiation oncology: a review of its current status and potential application for the radiotherapy workforce. Radiography. 2021;27:S63‐S68. 34493445 10.1016/j.radi.2021.07.012

[10] Atun R , Jaffray DA , Barton MB , et al. Expanding global access to radiotherapy. Lancet Oncol. 2015;16:1153‐1186. 26419354 10.1016/S1470-2045(15)00222-3

[11] Hindocha S , Zucker K , Jena R , et al. Artificial intelligence for radiotherapy auto‐contouring: current use, perceptions of and barriers to implementation. Clin Oncol. 2023;35:219‐226. 10.1016/j.clon.2023.01.014 36725406

[12] Peters LJ , O'sullivan B , Giralt J , et al. Critical impact of radiotherapy protocol compliance and quality in the treatment of advanced head and neck cancer: results from TROG 02.02. J Clin Oncol. 2010;28:2996‐3001. 20479390 10.1200/JCO.2009.27.4498

[13] Wuthrick EJ , Zhang Q , Machtay M , et al. Institutional clinical trial accrual volume and survival of patients with head and neck cancer. J Clin Oncol. 2015;33:156‐164. 25488965 10.1200/JCO.2014.56.5218 PMC4279235

[14] Kachnic LA , Winter K , Myerson RJ , et al. RTOG 0529: a phase 2 evaluation of dose‐painted intensity modulated radiation therapy in combination with 5‐fluorouracil and mitomycin‐c for the reduction of acute morbidity in carcinoma of the anal canal. Int J Radiat Oncol. 2013;86:27‐33. 10.1016/j.ijrobp.2012.09.023 PMC3619011 23154075

[15] Belkacemi Y , Colson‐Durand L , Fayolle‐Campana M , et al. A wake‐up call for routine morbidity and mortality review meeting procedures as part of a quality governance programs in radiation therapy departments: results of the PROUST survey. Pract Radiat Oncol. 2019;9:108‐114. 30268430 10.1016/j.prro.2018.09.004

[16] Vandewinckele L , Claessens M , Dinkla A , et al. Overview of artificial intelligence‐based applications in radiotherapy: recommendations for implementation and quality assurance. Radiother Oncol. 2020;153:55‐66. 32920005 10.1016/j.radonc.2020.09.008

[17] Hurkmans C , Bibault J‐E , Brock KK , et al. A joint ESTRO and AAPM guideline for development, clinical validation and reporting of artificial intelligence models in radiation therapy. Radiother Oncol. 2024;197:110345. 38838989 10.1016/j.radonc.2024.110345

[18] Sibolt P , Andersson LM , Calmels L , et al. Clinical implementation of artificial intelligence‐driven cone‐beam computed tomography‐guided online adaptive radiotherapy in the pelvic region. Phys Imaging Radiat Oncol. 2021;17:1‐7. 33898770 10.1016/j.phro.2020.12.004 PMC8057957

[19] Archambault Y , Boylan C , Bullock D , et al. Making on‐line adaptive radiotherapy possible using artificial intelligence and machine learning for efficient daily re‐planning. Med Phys Int J. 2020;8:77‐86.

[20] Varian Medical Systems . Ethos Algorithms Reference Guide. 2023, P1050581‐001‐A.

[21] Schreier J , Genghi A , Laaksonen H , Morgas T , Haas B . Clinical evaluation of a full‐image deep segmentation algorithm for the male pelvis on cone‐beam CT and CT. Radiother Oncol. 2020;145:1‐6. 31869676 10.1016/j.radonc.2019.11.021

[22] Ronneberger O , Fischer P , Brox T . U‐Net: convolutional networks for biomedical image segmentation. In: Navab N , ed. Medical Image Computing and Computer‐Assisted Intervention—MICCAI 2015. Springer International Publishing; 2015:234‐241.

[23] Milletari F , Navab N , Ahmadi S‐A , V‐Net: fully convolutional neural networks for volumetric medical image segmentation. 2016 Fourth International Conference on 3D Vision (3DV) 565‐571 (IEEE, Stanford, CA, USA, 2016). DOI: 10.1109/3DV.2016.79

[24] Brouwer CL , Steenbakkers RJHM , Bourhis J , Budach W , et al. CT‐based delineation of organs at risk in the head and neck region: DAHANCA, EORTC, GORTEC, HKNPCSG, NCIC CTG, NCRI, NRG Oncology and TROG consensus guidelines. Radiother Oncol. 2015;117:83‐90. 26277855 10.1016/j.radonc.2015.07.041

[25] Kong F‐MS , Ritter T , Quint DJ , et al. Consideration of dose limits for organs at risk of thoracic radiotherapy: atlas for lung, proximal bronchial tree, esophagus, spinal cord, ribs, and brachial plexus. Int J Radiat Oncol. 2011;81:1442‐1457. 10.1016/j.ijrobp.2010.07.1977 PMC3933280 20934273

[26] White J , Tail A , Arthur D , et al. RTOG breast cancer atlas for radiation therapy planning: consensus definitions. 2013.

[27] Jabbour SK , Hashem SA , Bosch W , et al. Upper abdominal normal organ contouring guidelines and atlas: a radiation therapy oncology group consensus. Pract Radiat Oncol. 2014;4:82‐89. 24890348 10.1016/j.prro.2013.06.004 PMC4285338

[28] Gay HA , Barthold HJ , O'meara E , et al. Pelvic normal tissue contouring guidelines for radiation therapy: a radiation therapy oncology group consensus panel atlas. Int J Radiat Oncol. 2012;83:e353‐e362. 10.1016/j.ijrobp.2012.01.023 PMC3904368 22483697

[29] Mir R , Kelly SM , Xiao Y , et al. Organ at risk delineation for radiation therapy clinical trials: global harmonization group consensus guidelines. Radiother Oncol. 2020;150:30‐39. 32504762 10.1016/j.radonc.2020.05.038

[30] Wright JL , Yom SS , Awan MJ , et al. Standardizing normal tissue contouring for radiation therapy treatment planning: an ASTRO consensus paper. Pract Radiat Oncol. 2019;9:65‐72. 30576843 10.1016/j.prro.2018.12.003

[31] Lin D , Lapen K , Sherer MV , et al. A systematic review of contouring guidelines in radiation oncology: analysis of frequency, methodology, and delivery of consensus recommendations. Int J Radiat Oncol. 2020;107:827‐835. 10.1016/j.ijrobp.2020.04.011 PMC8262136 32311418

[32] Mackay K , Bernstein D , Glocker B , Kamnitsas K , Taylor A . A review of the metrics used to assess auto‐contouring systems in radiotherapy. Clin Oncol. 2023;35:354‐369. 10.1016/j.clon.2023.01.016 36803407

[33] Sherer MV , Lin D , Elguindi S , et al. Metrics to evaluate the performance of auto‐segmentation for radiation treatment planning: a critical review. Radiother Oncol. 2021;160:185‐191. 33984348 10.1016/j.radonc.2021.05.003 PMC9444281

[34] Chlap P , Finnegan RNPPy . Processing library and analysis toolkit for medical imaging in python. J Open Source Softw. 2023;8:5374.

[35] Dice LR,. Measures of the amount of ecologic association between species. Ecology. 1945;26:297‐302.

[36] Nikolov S , Blackwell S , Zverovitch A , et al. Clinically applicable segmentation of head and neck anatomy for radiotherapy: deep learning algorithm development and validation study. J Med Internet Res. 2021;23:e26151. 34255661 10.2196/26151 PMC8314151

[37] Heimann T , Van Ginneken B , Styner MA , et al. Comparison and evaluation of methods for liver segmentation from CT datasets. IEEE Trans Med Imaging. 2009;28:1251‐1265. 19211338 10.1109/TMI.2009.2013851

[38] Groß W,. Grundzüge der Mengenlehre: von Felix hausdorff. Veitu. Co., Leipzig 1914. 476 S. M. 18. Monatshefte Für Math Phys. 1915;26:A34‐A35.

[39] Pedregosa F , Varoquaux G , Gramfort A , et al. Scikit‐learn: machine learning in python. Mach Learn PYTHON. 10.3389/fninf.2014.00014 PMC3930868 24600388

[40] Karimi D . Improving calibration and out‐of‐distribution detection in deep models for medical image segmentation. IEEE Trans Artif Intell. 2023;4:383‐397. 37868336 10.1109/tai.2022.3159510 PMC10586223

[41] Isaksson LJ , Summers P , Mastroleo F , et al. Automatic segmentation with deep learning in radiotherapy. Cancers. 2023;15:4389. 37686665 10.3390/cancers15174389 PMC10486603

[42] Chen W , Wang C , Zhan W , et al. A comparative study of auto‐contouring softwares in delineation of organs at risk in lung cancer and rectal cancer. Sci Rep. 2021;11:23002. 34836989 10.1038/s41598-021-02330-y PMC8626498

[43] Chen X , Sun S , Bai N , et al. A deep learning‐based auto‐segmentation system for organs‐at‐risk on whole‐body computed tomography images for radiation therapy. Radiother Oncol. 2021;160:175‐184. 33961914 10.1016/j.radonc.2021.04.019

[44] Radici L , Ferrario S , Borca VC , et al. Implementation of a commercial deep learning‐based auto segmentation software in radiotherapy: evaluation of effectiveness and impact on workflow. Life. 2022;12:2088. 36556455 10.3390/life12122088 PMC9782080

[45] Pera Ó , Martínez Á , Möhler C , et al. Clinical validation of siemens’ syngo.via automatic contouring system. Adv Radiat Oncol. 2023;8:101177. 36865668 10.1016/j.adro.2023.101177 PMC9972393

[46] Maduro Bustos LA , Sarkar A , Doyle LA , et al. Feasibility evaluation of novel AI‐based deep‐learning contouring algorithm for radiotherapy. J Appl Clin Med Phys. 2023;24:e14090. 37464581 10.1002/acm2.14090 PMC10647981

[47] Balagopal A , Kazemifar S , Nguyen D , et al. Fully automated organ segmentation in male pelvic CT images. Phys Med Biol. 2018;63:245015. 30523973 10.1088/1361-6560/aaf11c

[48] Liu Z , Liu X , Xiao B , et al. Segmentation of organs‐at‐risk in cervical cancer CT images with a convolutional neural network. Phys Med. 2020;69:184‐191. 31918371 10.1016/j.ejmp.2019.12.008

[49] Kalantar R , Lin G , Winfield JM , et al. Automatic segmentation of pelvic cancers using deep learning: state‐of‐the‐art approaches and challenges. Diagnostics. 2021;11:1964. 34829310 10.3390/diagnostics11111964 PMC8625809

[50] Heilemann G , Buschmann M , Lechner W , et al. Clinical implementation and evaluation of auto‐segmentation tools for multi‐site contouring in radiotherapy. Phys Imaging Radiat Oncol. 2023;28:100515. 38111502 10.1016/j.phro.2023.100515 PMC10726238

[51] Liao W , Luo X , He Y , et al. Comprehensive evaluation of a deep learning model for automatic organs‐at‐risk segmentation on heterogeneous computed tomography images for abdominal radiation therapy. Int J Radiat Oncol. 2023;117:994‐1006. 10.1016/j.ijrobp.2023.05.034 37244625

[52] Weston AD , Korfiatis P , Philbrick KA , et al. Complete abdomen and pelvis segmentation using U‐net variant architecture. Med Phys. 2020;47:5609‐5618. 32740931 10.1002/mp.14422

[53] Chung SY , Chang JS , Choi MS , et al. Clinical feasibility of deep learning‐based auto‐segmentation of target volumes and organs‐at‐risk in breast cancer patients after breast‐conserving surgery. Radiat Oncol. 2021;16:44. 33632248 10.1186/s13014-021-01771-z PMC7905884

[54] Johnston N , De Rycke J , Lievens Y , et al. Dose‐volume‐based evaluation of convolutional neural network‐based auto‐segmentation of thoracic organs at risk. Phys Imaging Radiat Oncol. 2022;23:109‐117. 35936797 10.1016/j.phro.2022.07.004 PMC9352974

[55] Mao W , Riess J , Kim J , et al. Evaluation of auto‐contouring and dose distributions for online adaptive radiation therapy of patients with locally advanced lung cancers. Pract Radiat Oncol. 2022;12:e329‐e338. 35219879 10.1016/j.prro.2021.12.017

[56] Zhang F , Wang Q , Yang A , et al. Geometric and dosimetric evaluation of the automatic delineation of organs at risk (OARs) in non‐small‐cell lung cancer radiotherapy based on a modified dense net deep learning network. Front Oncol. 2022;12:861857. 35371991 10.3389/fonc.2022.861857 PMC8964972

[57] Ye X , Guo D , Ge J , et al. Comprehensive and clinically accurate head and neck cancer organs‐at‐risk delineation on a multi‐institutional study. Nat Commun. 2022;13:6137. 36253346 10.1038/s41467-022-33178-z PMC9576793

[58] D'aviero A , Re A , Catucci F , et al. Clinical validation of a deep‐learning segmentation software in head and neck: an early analysis in a developing radiation oncology center. Int J Environ Res Public Health. 2022;19:9057. 35897425 10.3390/ijerph19159057 PMC9329735

[59] Nielsen CP , Lorenzen EL , Jensen K , et al. Consistency in contouring of organs at risk by artificial intelligence vs oncologists in head and neck cancer patients. Acta Oncol. 2023;62:1418‐1425. 37703300 10.1080/0284186X.2023.2256958

[60] Costea M , Zlate A , Durand M , et al. Comparison of atlas‐based and deep learning methods for organs at risk delineation on head‐and‐neck CT images using an automated treatment planning system. Radiother Oncol. 2022;177:61‐70. 36328093 10.1016/j.radonc.2022.10.029

[61] Samarasinghe G , Jameson M , Vinod S , et al. Deep learning for segmentation in radiation therapy planning: a review. J Med Imaging Radiat Oncol. 2021;65:578‐595. 34313006 10.1111/1754-9485.13286

[62] Liu P , Sun Y , Zhao X , Yan Y . Deep learning algorithm performance in contouring head and neck organs at risk: a systematic review and single‐arm meta‐analysis. Biomed Eng OnLine. 2023;22:104. 37915046 10.1186/s12938-023-01159-y PMC10621161

[63] Guzene L , Beddok A , Nioche C , et al. Assessing interobserver variability in the delineation of structures in radiation oncology: a systematic review. Int J Radiat Oncol. 2023;115:1047‐1060. 10.1016/j.ijrobp.2022.11.021 36423741

[64] Roach D , Holloway LC , Jameson MG , et al. Multi‐observer contouring of male pelvic anatomy: highly variable agreement across conventional and emerging structures of interest. J Med Imaging Radiat Oncol. 2019;63:264‐271. 30609205 10.1111/1754-9485.12844

[65] Casati M , Piffer S , Calusi S , et al. Clinical validation of an automatic atlas‐based segmentation tool for male pelvis CT images. J Appl Clin Med Phys. 2022;23:e13507. 35064746 10.1002/acm2.13507 PMC8906199

[66] Liu H , Amaloo C , Sintay B , Wiant D . Dosimetric effects due to inter‐observer variability of organ contouring when utilizing a knowledge‐based planning system for prostate cancer. Int J Med Phys Clin Eng Radiat Oncol. 2021;10:47‐58.

[67] Jones MP , Martin J , Foo K , Estoesta P , Holloway L , Jameson M . The impact of contour variation on tumour control probability in anal cancer. Radiat Oncol. 2018;13:97. 29776418 10.1186/s13014-018-1033-y PMC5960192

[68] Tsang Y , Hoskin P , Spezi E , et al. Assessment of contour variability in target volumes and organs at risk in lung cancer radiotherapy. Tech Innov Patient Support Radiat Oncol. 2019;10:8‐12. 32095541 10.1016/j.tipsro.2019.05.001 PMC7033767

[69] Arculeo S , Miglietta E , Nava F , et al. The emerging role of radiation therapists in the contouring of organs at risk in radiotherapy: analysis of inter‐observer variability with radiation oncologists for the chest and upper abdomen. Cancer Med Sci. 2020;14. 10.3332/ecancer.2020.996 PMC7032938 32153651

[70] Choi MS , Chang JS , Kim K , et al, Shin KH , Kim YB . Assessment of deep learning‐based auto‐contouring on interobserver consistency in target volume and organs‐at‐risk delineation for breast cancer: implications for RTQA program in a multi‐institutional study. Breast. 2024;73:103599. 37992527 10.1016/j.breast.2023.103599 PMC10700624

[71] Mccall R , Maclennan G , Taylor M , et al. Anatomical contouring variability in thoracic organs at risk. Med Dosim. 2016;41:344‐350. 27839589 10.1016/j.meddos.2016.08.004

[72] Mcwilliam A , Lee L , Harris M , et al. Benefit of using motion compensated reconstructions for reducing inter‐observer and intra‐observer contouring variation for organs at risk in lung cancer patients. Radiother Oncol. 2018;126:333‐338. 29221648 10.1016/j.radonc.2017.11.021

[73] Van Der Veen J , Gulyban A , Willems S , Maes F , Nuyts S . Interobserver variability in organ at risk delineation in head and neck cancer. Radiat Oncol. 2021;16:120. 34183040 10.1186/s13014-020-01677-2 PMC8240214

[74] Wong J , Fong A , Mcvicar N , et al. Comparing deep learning‐based auto‐segmentation of organs at risk and clinical target volumes to expert inter‐observer variability in radiotherapy planning. Radiother Oncol. 2020;144:152‐158. 31812930 10.1016/j.radonc.2019.10.019

[75] Podobnik G , Ibragimov B , Peterlin Pž , Strojan Pž , Vrtovec Tž . vOARiability: interobserver and intermodality variability analysis in OAR contouring from head and neck CT and MR images. Med Phys. 2024;51:2175‐2186. 38230752 10.1002/mp.16924

[76] Vaassen F , Hazelaar C , Vaniqui A , et al. Evaluation of measures for assessing time‐saving of automatic organ‐at‐risk segmentation in radiotherapy. Phys Imaging Radiat Oncol. 2020;13:1‐6. 33458300 10.1016/j.phro.2019.12.001 PMC7807544

[77] Wang C , Zhu X , Hong JC , Zheng D . Artificial intelligence in radiotherapy treatment planning: present and future. Technol Cancer Res Treat. 2019;18:153303381987392. 10.1177/1533033819873922 PMC6732844 31495281

[78] Maes D , Gates EDH , Meyer J , et al. Framework for radiation oncology department‐wide evaluation and implementation of commercial artificial intelligence autocontouring. Pract Radiat Oncol. 2024;14:e150‐e158. 37935308 10.1016/j.prro.2023.10.011

[79] Jones S , Thompson K , Porter B , et al. Automation and artificial intelligence in radiation therapy treatment planning. J Med Radiat Sci. 2024;71:290‐298. 37794690 10.1002/jmrs.729 PMC11177028

[80] Andersson J , Nyholm T , Ceberg C , et al. Artificial intelligence and the medical physics profession—a Swedish perspective. Phys Med. 2021;88:218‐225. 34304045 10.1016/j.ejmp.2021.07.009

[81] Altman MB , Kavanaugh JA , Wooten HO . A framework for automated contour quality assurance in radiation therapy including adaptive techniques. Phys Med Biol. 2015;60:5199‐5209. 26083863 10.1088/0031-9155/60/13/5199

[82] Baroudi H , Brock KK , Cao W , et al. Automated contouring and planning in radiation therapy: what is ‘clinically acceptable’?. Diagnostics. 2023;13:667. 36832155 10.3390/diagnostics13040667 PMC9955359

