# Supplementary material for: Geometric and dosimetric evaluation of a commercial AI auto‐contouring tool on multiple anatomical sites in CT scans
Source: J Appl Clin Med Phys. 2025 Mar 17;26(6):e70067. doi: 10.1002/acm2.70067 (PMC12148769; doi:10.1002/acm2.70067)
Supplement: Supplementary file 1 — Supporting information [file ACM2-26-e70067-s001.docx]

# Supplementary material

**Table S1.** List of structures included in Ethos AI auto-contouring models for each anatomical site and treatment cohort.

| **Anatomical site** | **Treatment cohort** | **Structures** |
| --- | --- | --- |
| Pelvis | Anus | Bladder |
|  |  | Femoral head (L/R) |
|  |  | Rectum |
|  |  | Sigmoid colon |
|  |  | Small bowel |
|  | Bladder | Femoral head (L/R) |
|  |  | Rectum |
|  |  | Sigmoid colon |
|  |  | Small bowel |
|  | Gynae (LN) | Bladder |
|  |  | Femoral head (L/R) |
|  |  | Rectum |
|  |  | Sigmoid colon |
|  |  | Small bowel |
|  |  | Kidneys (L/R) |
|  |  | Liver |
| Abdomen | Pancreas | Small bowel |
|  |  | Kidneys (L/R) |
|  |  | Liver |
|  |  | Duodenum |
|  |  | Stomach |
|  |  | Spinal cord |
|  | Liver SBRT | Kidneys (L/R) |
|  |  | Liver |
|  |  | Duodenum |
|  |  | Stomach |
|  |  | Spinal cord |
|  |  | Oesophagus |
|  |  | Heart |
|  |  | Chestwall |
| Thorax | Oesophagus | Carina |
|  |  | Lungs (L/R) |
|  |  | Heart |
|  |  | Spinal cord |
|  |  | Liver |
|  |  | Kidneys (L/R) |
|  | Mediastinum | Carina |
|  |  | Lungs (L/R) |
|  |  | Heart |
|  |  | Spinal cord |
|  |  | Breasts (L/R) |
|  | Lung (SBRT) | Carina |
|  |  | Lungs (L/R) |
|  |  | Heart |
|  |  | Spinal cord |
|  |  | Oesophagus |
|  |  | Chestwall |
|  | Breast (left) | Breasts (L/R) |
|  |  | Liver |
|  |  | Heart |
|  |  | Lungs (L/R) |
|  |  | Oesophagus |
|  |  | Spinal cord |
|  |  | Thyroid |
|  |  | Trachea |

| **Anatomical site** | **Treatment cohort** | **Structures** |
| --- | --- | --- |
| Head and Neck | Naso-, oro-, hypo-pharynx | Brain |
|  |  | Brainstem |
|  |  | Cochleae (L/R) |
|  |  | Eye (L/R) |
|  |  | Larynx |
|  |  | Lens (L/R) |
|  |  | Lips |
|  |  | Mandible |
|  |  | Oesophagus |
|  |  | Optic chiasm |
|  |  | Optic nerve (L/R) |
|  |  | Oral cavity |
|  |  | Parotid gland (L/R) |
|  |  | Pharyngeal constrictor muscle (sup., mid., inf.) |
|  |  | Spinal cord |
|  |  | Submandibular gland (L/R) |
| All | All | Body |

**Table S2.** Description of automated processing used to harmonise the definitions of Ethos AI auto-contours and local contouring practices. This was completed to facilitate quantitative comparisons.

| **Structure** | **Local definition** | **Ethos AI definition** | **Processing** | **Comparison** |
| --- | --- | --- | --- | --- |
| Small bowel | Bowel bag (peritoneal cavity) near treatment target | Bowel loops (duodenum, jejunum, and ileum) visible within scan | Ethos AI contour cropped to sup/inf limits of manual contours | Relative volume of cropped Ethos AI contour within manual contour |
| Sigmoid colon | Sigmoid colon near treatment target | Sigmoid colon visible within scan |  |  |
| Bronchial tree / carina | Carina (ridge of cartilage between two main bronchi) | Bronchial tree, including distal 2 cm of trachea and main bronchi | No change | Relative volume of carina within bronchial tree |
| Chest wall | Ipsilateral region of chest wall near treatment target  Thickness 2.5 cm,  sup/inf limits of 3 cm from PTV | Entire chest wall (contra- and ipsilateral) visible within scan  Thickness 2.0 cm | No change | Only dosimetric evaluation |
| Trachea | Region of structure near treatment target | Entire structure visible within scan | Ethos AI contour cropped to sup/inf limits of manual contours | All geometric comparisons |
| Oesophagus |  |  |  |  |
| Spinal cord |  |  |  |  |
| Larynx | Triangular region extending from caudal extent of hyoid and ending at the cricoid | “Glottis” structure, extending from cranial edge of arytenoid cartilages to the caudal extent of thyroid cartilage | No change | All geometric comparisons |

| **Figure S1 (overleaf).** Distribution of geometric measures of similarity between manual contours and Ethos AI auto-contours, separated by structure, represented as box plots. For each structure the total number of patients included in the analysis is shown in brackets after the name, and the mean ± standard deviation for each structure and measure is shown above the plot area. Outliers, indicated by open circles, are defined as any value more than 1.5 × inter‑quartile range away from the first or third quartile. |
| --- |


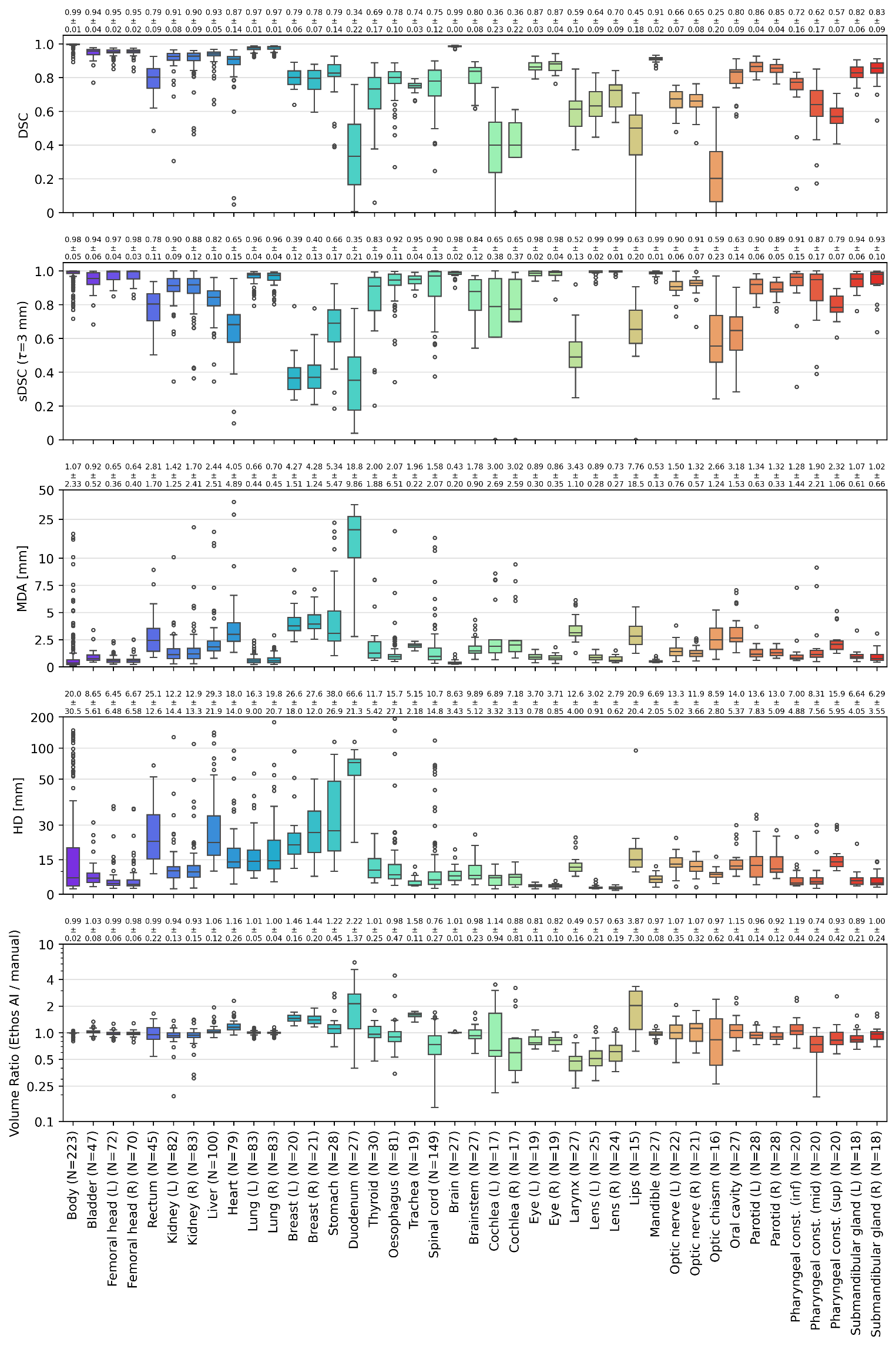


| 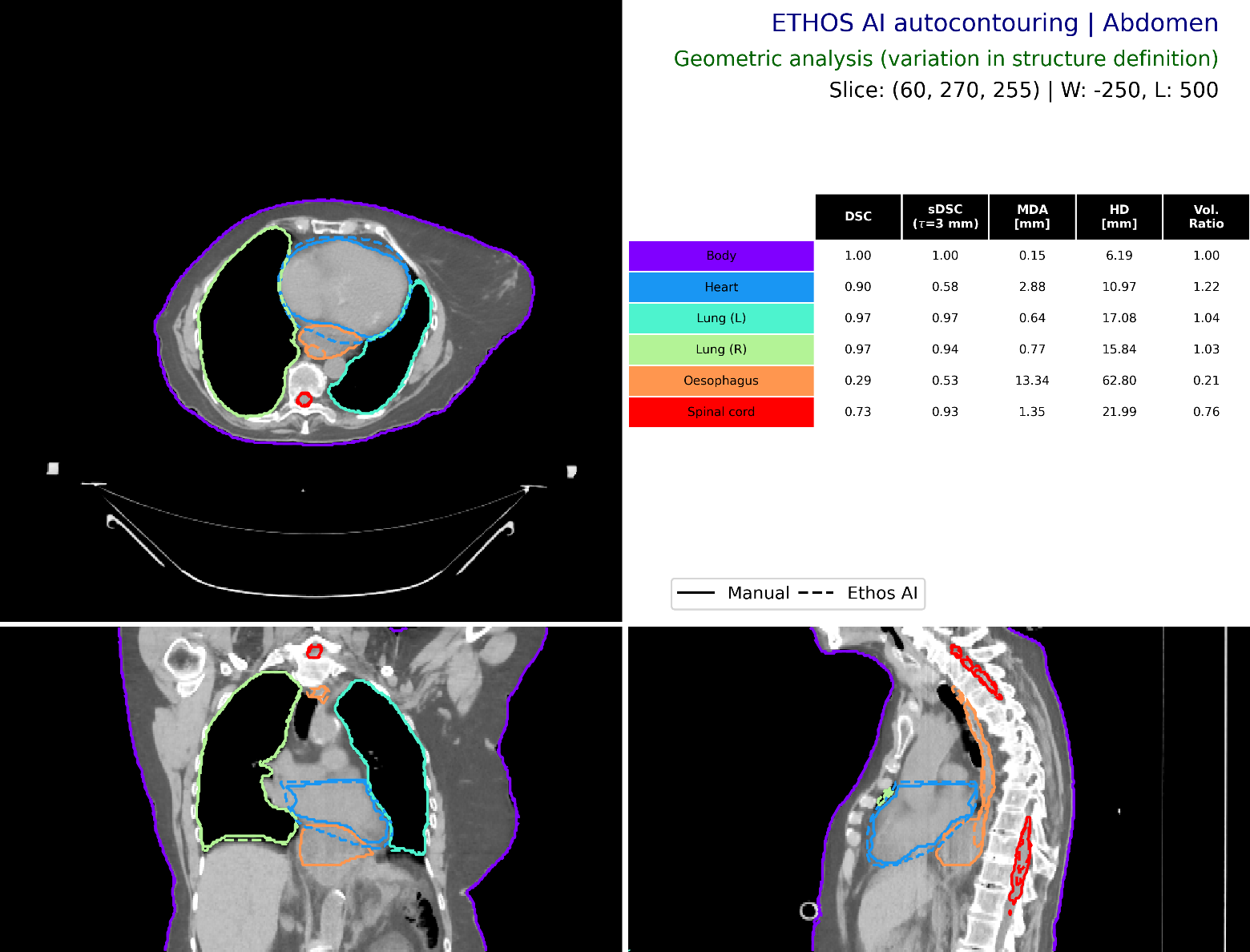 |
| --- |
| **Figure S2.** Representative example case, demonstrating a situation where the manual oesophagus contour includes the proximal stomach. This decision may have been made during planning to ensure sparing of healthy tissue in this region. The Ethos AI auto-contour includes only the anatomical oesophagus, and therefore geometric agreement is low (DSC=0.29, HD=62.8 mm). Other structures were accurately contoured. |

| 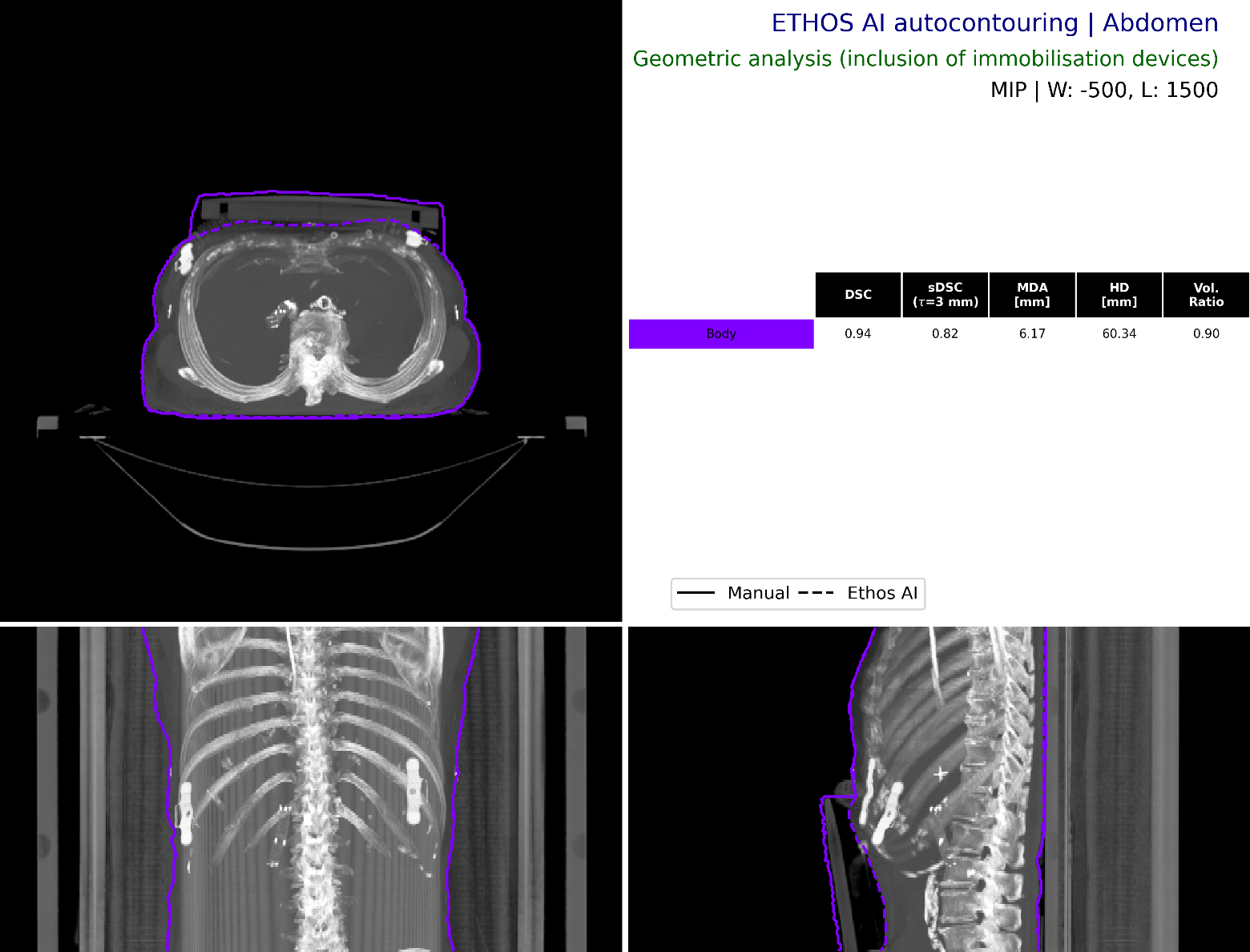 |
| --- |
| **Figure S3.** Representative example case, demonstrating a situation in which the abdominal compression device is included in the “manual” contour set. Note that the external body contour is automatically generated in both the Eclipse TPS (during creation of the manual contour set) and the Ethos TPS. |

| 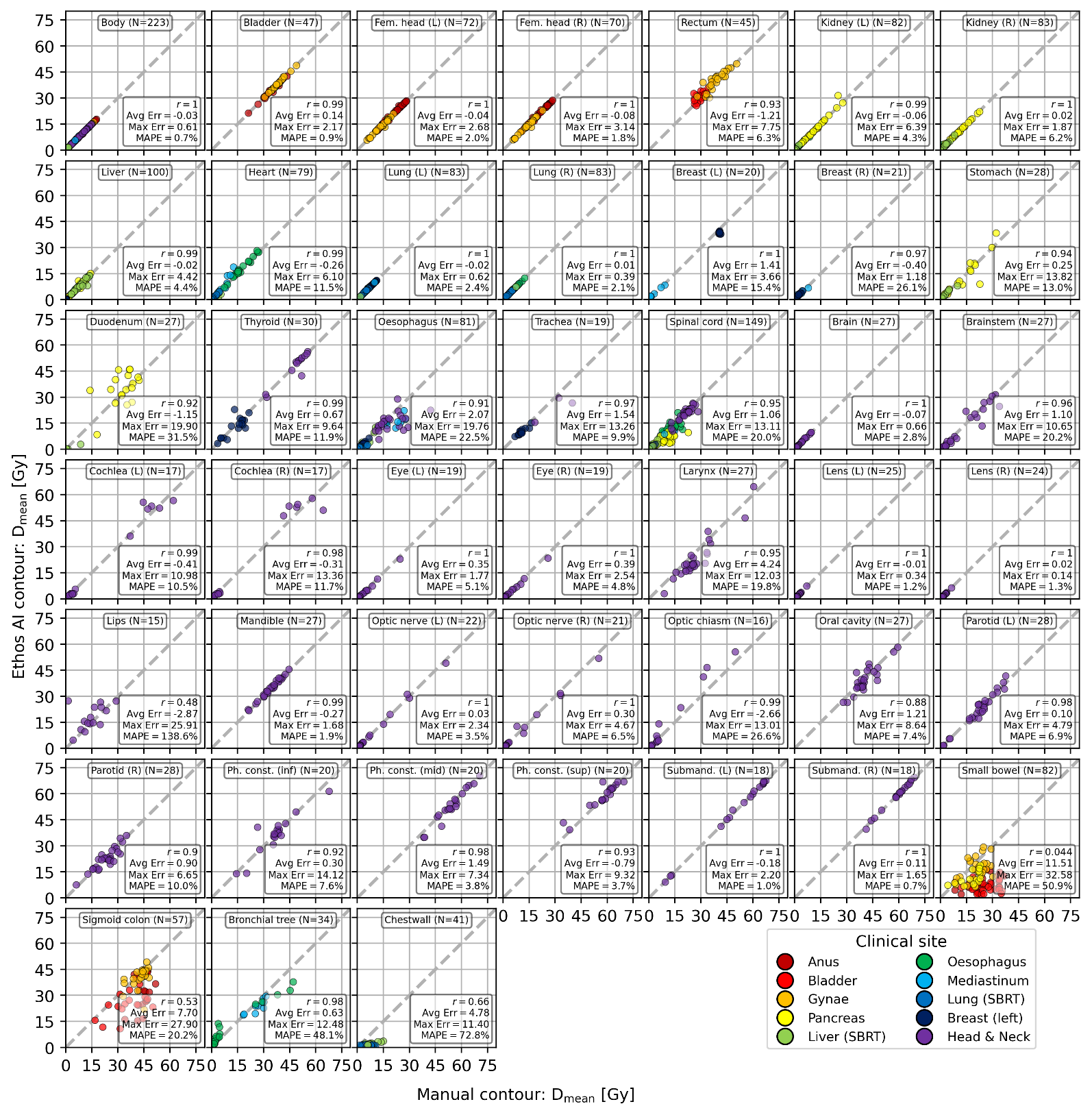 |
| --- |
| **Figure S4.** Dose-volume metrics derived from the manual contour (x-axis) are compared with those from the Ethos AI auto-contours (y-axis). The mean dose (D_mean_) is calculated from the clinical dose grid. The line of unity is shown (grey dashed), and for each structure the number of patients included in the analysis is shown in brackets after the name. |

| 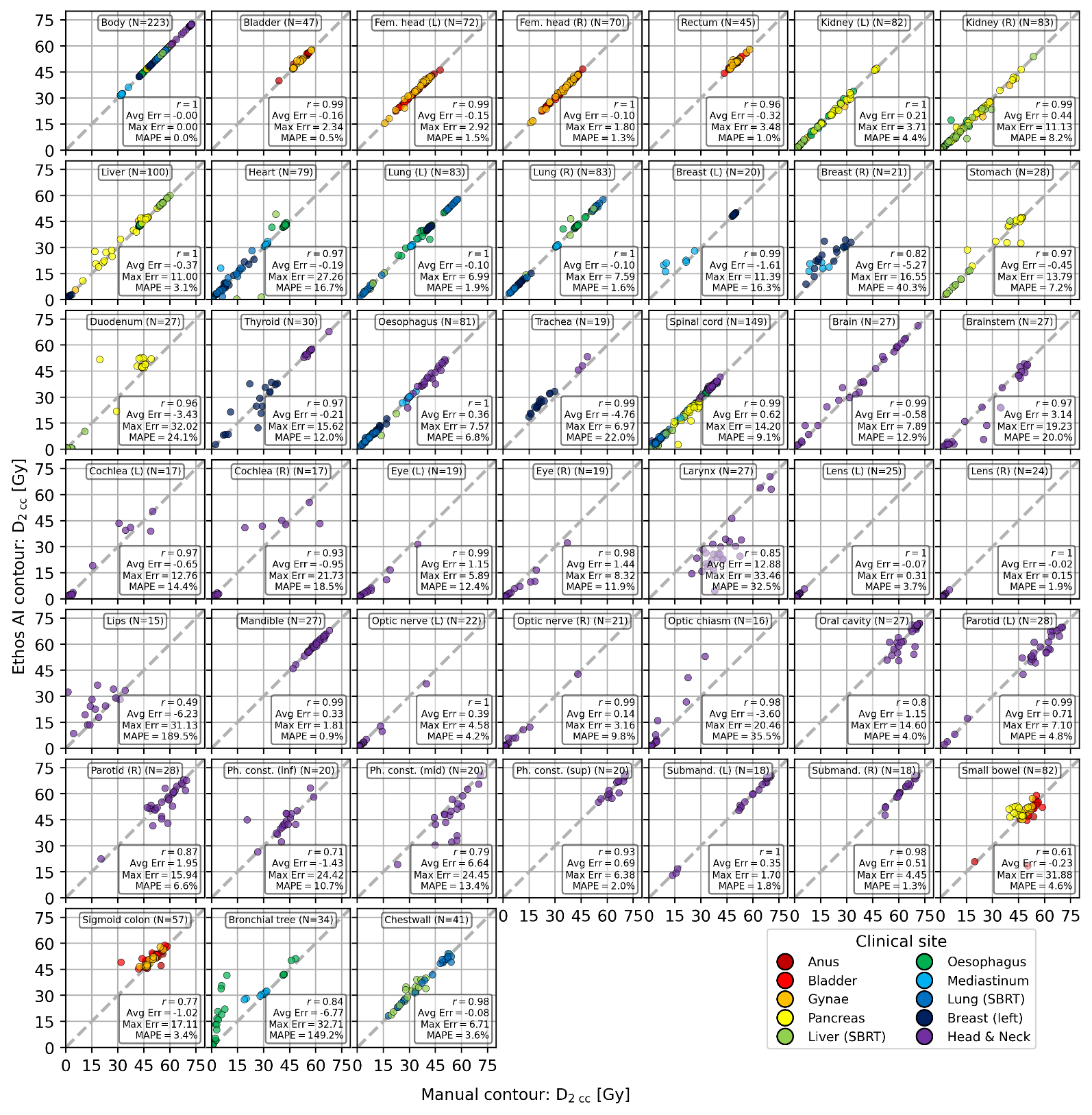 |
| --- |
| **Figure S5.** Dose-volume metrics derived from the manual contour (x-axis) are compared with those from the Ethos AI auto-contours (y-axis). The dose to the hottest 2 cm^3^ (D_2 cc_) is calculated from the clinical dose grid. The line of unity is shown (grey dashed), and for each structure the number of patients included in the analysis is shown in brackets after the name. |

| 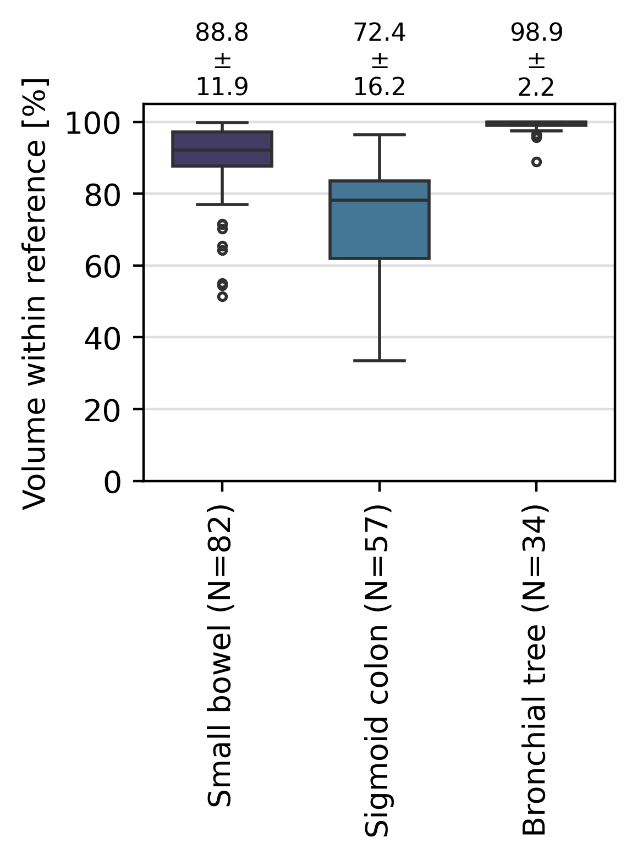 | **Figure S6.** Distributions of the relative volume of the Ethos AI auto-contour within the manual contour for the small bowel and sigmoid colon, and vice versa for the bronchial tree and chestwall. Refer to Table S2 for more information.  For each structure the total number of patients included in the analysis is shown in brackets after the name, and the mean ± standard deviation for each structure and measure is shown above the plot area. Outliers, indicated by open circles, are defined as any value more than 1.5 × inter‑quartile range away from the first or third quartile. |
| --- | --- |
